# Qualitative mucin disorders in patients with primary Sjögren's syndrome: a literature review

**DOI:** 10.4317/medoral.23996

**Published:** 2020-11-28

**Authors:** Massimo Fusconi, Francesca Candelori, Lior Weiss, Annachiara Riccio, Roberta Priori, Rita Businaro, Linda Mastromanno, Isotta Musy, Marco de Vincentiis, Antonio Greco

**Affiliations:** 1 MD, Specialist in ENT, Division of Otorhinolaryngology; Department of Sensory Organs, University of Rome “Sapienza”, Italy; 2 MD, Resident in ENT, Division of Otorhinolaryngology; Department of Sensory Organs, University of Rome “Sapienza”, Italy; 3 MD, Visiting doctor, Division of Otorhinolaryngology; Department of Sensory Organs, University of Rome “Sapienza”, Italy; 4 MD, Specialist in Allergy and Clinical Immunology, Rheumatology Unit, Department of Internal Medicine and Medical Specialties, University of Rome “Sapienza”, Italy; 5 MD, Specialist Clinical Pathology, Professor of Human Anatomy; Department of Medico-Surgical Sciences and Biotechnologies, University of Rome “Sapienza”, Italy; 6 MD, Visiting doctor, Rheumatology Unit, Department of Internal Medicine and Medical Specialties, University of Rome “Sapienza”, Italy; 7 MD, Specialist in ENT, ENT Police Doctor, Italy; 8 MD, Specialist in ENT, Professor in ENT, Department of Oral and Maxillofacial Science, University of Rome “La Sapienza”, Italy.; 9 MD, Specialist in ENT, Professor in ENT, Division of Otorhinolaryngology, Department of Sensory Organs, University of Rome “Sapienza”, Italy

## Abstract

**Background:**

It is a common opinion that Primary Sjögren Syndrome (pSS) damages the exocrine glands and determines the reduction of secreted saliva, some studies show that there are qualitative anomalies of the mucins produced in saliva, including MUC7, MUC5B, MUC1. The purpose of this study is to trace all the information useful to establish whether there is a qualitative or quantitative defect of the mucins in the pSS.

**Material and Methods:**

We reviewed the literature by looking for publications relevant to the topic in electronic databases. Sixteen articles met the search criteria. The studies were divided into two categories, those that studied the rheological characteristics of the saliva and those that studied the structural and / or metabolism modifications of the muciparous cells in the salivary glands.

**Results:**

in Patients with pSS, xerostomia and the reduction of salivary spinnbarkeit are only partially related to the reduction of the unstimulated salivary flow. In pSS, pathological alterations of mucins’ chemical-physical properties prevail as a cause of the clinical characteristics. Moreover, in pSS there are structural and metabolism changes in salivary glands’ muciparous cells.

**Conclusions:**

There is much evidence that supports the presence of qualitative alterations in the saliva’s rheological properties in Patients with pSS, and these are the main cause, more than the reduction of the unstimulated salivary flow, of the disease clinical characteristics - dry mouth and complications in the oral cavity. Therefore we propose to add to the classification criteria of pSS also a qualitative test of salivary glycoproteins.

** Key words:**Primary Sjögren's syndrome, mucin, MUC7, MUC5B, MUC1, sulphate oligosaccharides.

## Introduction

Primary Sjögren's Syndrome (pSS) is an autoimmune disease that predominantly affects the exocrine glands compromising their secreting functions, resulting in xerophthalmia if it affects the lacrimal glands and/or xerostomia if it affects the salivary glands and/or genital dryness if it affects the glands of the vaginal mucosa. Other symptoms are arthralgia, myalgia, fatigue, depression, cutaneous vasculitis. The salivary glands produce saliva composed by 99% water, 0.5% organic and inorganic compounds and only 0.5% protein. Mucins are the most abundant proteins: MUC7, is a low molecular weight glycoprotein (200-300 kDa), in the sol phase, which forms the external saliva fraction, moved by the ciliary mucous transport. MUC5B in the other hand is a high molecular weight glycoprotein (> 1000 kDa) gelling agent, which forms the lining film of the mucous membranes and which forms the protective shield of the epithelium (1-4).

MUC1 is a glycoprotein (250-500 kDa) which through a transmembrane domain is linked to the cell membranes in the apical portion of the epithelium of the oral mucosa and salivary glands (5). It has a protective function, by binding to pathogens, and hydration function of the oral surface, but it is function in the oral environment remains uncertain.

The mucin is composed of various amino acids skeleton: serine, alanine, proline, glycine and threonine, which allow a N and O glycosylation (1,2). The O-glycans, represent about 80% of the mucin's molecular mass, (6) are mainly N-acetyl-galactosamine and other sugar residues such as galactose, fucose, sialic acid (SA) and sulphate (7).

The result is a central linear protein conFiguration with oligosaccharide side chains with a "bottlebrush" architecture flanked by glycid globular domains (8).

Mucins have mainly negative electric charge, hydrophilicity, conferred by SO3 groups, by the fucose and sialic acid hydroxyl group ¯OH, and by the carboxylic group -COOH of sialic acid (9). Mucins also contain hydrophobic groups which give them amphiphilic qualities, leading them to adhere to both hydrophobic and hydrophilic surfaces, enhancing their protection and lubrication capabilities (8). The chemical and physical characteristics of the mucins allow them to protect the oral cavity and pharynx, as they adhere, moisten and lubricate the oral mucosa and teeth, inhibiting bacterial growth and promoting their removal into the sol layer. In addition, the molecule is sTable during temperature and pH fluctuations induced by food (10).

It is a common opinion, in clinical practice, that pSS damages salivary glands and determines a reduction in the amount of saliva secreted, in particular, the clinical characteristics of the disease are considered to be in close relationship with salivary hypo-inflow. A confirm of that is given by the clinical practice’ criteria for the diagnosis of pSS, recommended by the American College of Rheumatology / European League, which advocates the use of Sialometry methods to measure the salivary flow, where a flow of ≤0.1 ml / min is considered pathological (Table 1) (11).

On the contrary, tests which evaluate changes in saliva quality, in particular in the mucins component, are not recommended yet. The purpose of this study is to trace all the information and evidence useful to establish whether in the pSS there is a quantitative and/or qualitative defect of the mucins which cause the clinical aspects of the disease. In case of qualitative defects, we would propose adding a qualitative test to the classification criteria of the pSS. For this purpose a literature review was conducted.

**Table 1:**
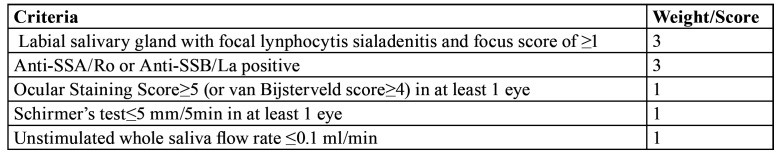
ACR / EULAR Classification Criteria for pSS.

## Material and Methods

- Search strategy

In order to provide the best evidence available to establish whether in the pSS there is a qualitative or quantitative defect in saliva, we have carried out a literature review looking for the publications relevant to the subject in the electronic databases.

One first group of keywords included:

Primary Sjögren's syndrome and Xerostomia or/and hyposalivation

Combined, in un second step, with one the following terms: 1. Mucin; 2. MUC7; 3. MUC5B; 4. MUC1

The electronic search was finished on April 30, 2020, no limitation on the publication date was imposed. The inclusion criteria have been: only texts in English were taken in consideration, only manuscripts that contained the first group of keywords (Primary Sjögren's syndrome and Xerostomia or/and hyposalivation) a complete of 759 manuscripts; in a second step, all manuscripts that did not contain at least one term from the second group were excluded from the review (mucin or MUC1 or MUC5B or MUC7) The manuscripts’ abstracts were read and if they provided useful information, then the entire article was analyzed. Manuscripts containing only generic references were also excluded.

**Figure 1:**
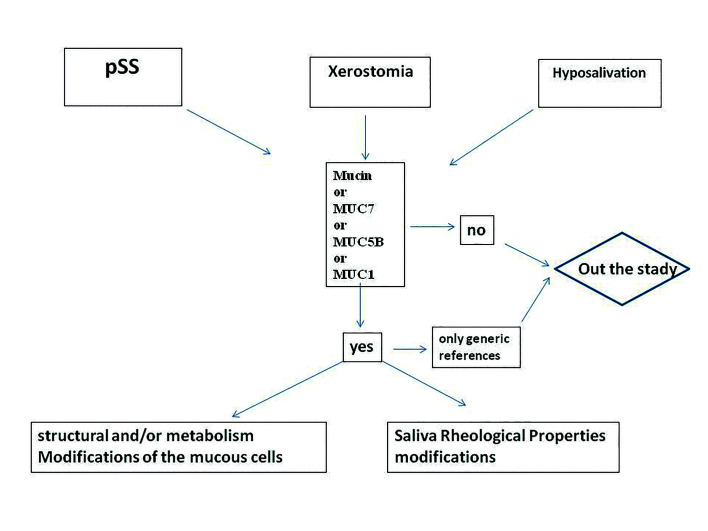
Revision: decision algorithm.

Only articles that correlated the rheological anomalies of mucins with the etiopathogenesis of the pSS and/or articles that correlated the structural or metabolism modifications of the minor salivary glands’ (MSG) mucous acinar cells with the etiopathogenesis of pSS were admitted. Revisions were also used but only those in which the author compared his previous studies with experiences of other researchers (Fig. 1).

## Results

19 studies contained the combination of one of the keywords: Primary Sjögren Syndrome plus Xerostomia or hyposalivation with Mucin or MUC7 or MUC5B or MUC1. Sixteen articles met the criteria and entered the final phase of critical evaluation, three articles were not admitted as the articles contained only generic references on the problem. The studies were divided into two categories, those that studied the rheological characteristics of saliva as a function of its glycoprotein mucins (8 articles) and those that studied the structural and/or metabolism modifications of the mucous cells (8 articles) in the minor salivary glands or submaxillary or sublingual glands.

- Evidence, traced in the literature, of the qualitative anomalies of the Saliva Rheological Properties and Mucin Glycosylation

In 8 articles, the pathological modifications of the mucins chemical and physical properties were studied: namely viscosity, elasticity, stickiness.

The first article, in chronological order traced, was written by Nederfors T *et al*. In 2000, (12) the Authors state states that the terms xerostomia and hyposalivation, in patients with salivary hyposecretion compreso la pSS, express two different concepts "xerostomia denotes the subjective feeling, the symptom, of dry mouth, while hyposalivation denotes an objective sign, a decreased saliva flow rate". Previously other authors had expressed the same concept (13). Nederfors hypothesizes how the change in the protein component of saliva may be the basis of xerostomia, thus distinguishing between qualitative or quantitative variation of the mucin.

In 2007 Alliende C *et al*. (14) made a study on sulfur glycoprotein MUC5B inside MSG; this study showed that there is a reduction, not statistically significant, of the mRNA of MUC5B and of the glycoprotein itself, on the other hand there was a significant decrease in total sulphate oligosaccharides and in the sulfurization process. The author first indicated the Golgi complex (GC) as a possible site of the MUC5B composition error, where sulphatases whose anomalous function would determine the error, would be present (15).

He also hypothesized that xerostomia is not related to reduced unstimulated salivary flow (USF) but rather to the reduced sulphation of MUC5B and other mucins.

In 2010, Pramanik R. *et al*. have performed a study on the presence of proteins, MUC5B and MUC7 in different areas of the oral cavity in patients with salivary hyposecretion, some of whom had pSS, concluding that "the surfaces of the oral mucosa in patients with xerostomia can retain MUC5B and other salivary proteins, although the functional integrity of these proteins is uncertain." Also these authors therefore hypothesize a qualitative defect in the mucin (16).

In 2013 Castro I., author of several experimental studies on saliva, displayed in one of her reviews the characteristics of MUC5B inside the acinar cells of MSG and highlighted a reduced glycosylation of this mucin in post-translational compartments, including the endoplasmic reticulum, the GC and secretory granules and in particular a reduction of sulphate oligosaccharides. All patients complained of xerostomia which correlated with sulfurization deficiency rather than USF (Castro *et al*., 2013) (9). In 2015 Chaudhury N.M.A. *et al*. carried out a study partly similar to that of Alliende C *et al*. (14). The study enrolled patients with xerostomia and reduced spinnbarkeit of saliva some of whom had pSS. The study showed that: the concentrations of MUC5B and MUC7 were generally normal or even higher in those patients, compared to the sample, but showed a reduction of sialic acid and oligosaccharides sulphates and the same patients had an average USF below the normal threshold value, these anomalies were present mainly pSS patients (2). Therefore, for the first time, it is hypothesized that the reduction of mucosal hydration is related to the deficiency of sialic acid in addition to the already known deficiency of sulfated oligosaccharides.

In 2016, Chaudhury N.M.A. published an interesting study on the biochemistry of MUC5B and MUC7 compared to the rheological properties of saliva in pSS patients. This analysis suggested that the reduction of the USF, the residual mucosal saliva and the spinnbarkeit were related to the biochemical modifications of the mucins. Specifically: there was no decrease in the amount of MUC5B and MUC7 glycoproteins, but the average ratio between Mucin Glycan/Protein was greatly reduced as was the sialization of mucins. The author has identified the defect in the O-glycan sialization deficiency of nucleus 2. Glycosylation is 80% of the mucins mass, therefore deglycosylation according to the Authors determines "A reduction in sialic acid, as shown here in pSS, has a range of potential effects on the physical rheological properties of saliva as they relate to hydration and lubrication" (17).

In 2019 Gallo A. *et al*. Studied the miRNA-mediated mechanisms involved in mucin O-glycosylation, highlighting that, there are several upregulated miRNAs, all refer to genes involved in mucin O-glycosylation pathway. In particular, down-regulation concerns the genes responsible for glycosylation of MUC7 (18).

- Evidence, traced in literature, of Structural and/or Metabolism modifications of Mucous Acinar Cells in the Salivary Glands

MUC1

In a manuscript written by Pramanik R. [2010] *et al*. for the first time in Patients with pSS, quantitative variations of MUC1 were demonstrated. Lower quantities of MUC1 were found in salivary specimens collected from the anterior portion of the tongue epithelium (16).

In 2019 Culp D.J. *et al*. have shown that, in patients with pSS, MUC1 is present in the deeper cells layers of the hard palate’s keratinized epithelium, unlike the non-keratinized epithelium of the lips, where MUC1 is present in the apical layers and much less in the basal ones. This anomalous distribution, resulting from intracellular down regulation, would be the basis of xerostomia (19). Therefore the manuscript supports the findings of Pramanik’s study of non-homogeneous distribution of MUC1 in the mouth in pSS Patients (16).

In 2020 Castro I. came to different conclusions than previous authors. In MSG of patients with pSS there is an overexpression of MUC1 induced by cytokines, and not by a down-regulation, (19) the cytokines promote endoplasmic reticulum stress and consequent accumulation of MUC1. Castro I. therefore supports the hypothesis of an adjuvant proinflammatory role in the relationship between cytokines and MUC1, MUC5B, MUC7 (20).

cellular exocytosis

The assembly of the membrane fusion system, essential for cellular exocytosis, involves several protein families like SNAREs, Rab, GTPases, and Sec1 / Munc-18, as the following studies have shown.

The subfamily of Rab3 proteins is composed by 4 isoforms (A - D) (Smith *et al*., 1985) (21). In the cells of the exocrine glands, such as the pancreas, the parotid and the lacrimal glands, Rab3D represents the predominant isoform, in these glands it facilitates the exocytosis of many elements, such as secretory mucins.

In 2011 Bahamondes A. *et al*. analyzed the expression and localization of Rab3D in MSG. The authors demonstrated a significant decrease in Rab3D at the cell apex but a normal RNAm expression (22). These observations confirm the idea that the lack of Rab3D at the cell apex negatively affects the fusion of the granules with the plasma membrane with the consequent accumulation of MUC7 in the glandular acini in the basolateral portion. According to the authors, the phenomenon has two possible, but contradictory, explanations: the accumulation can be caused by an increase in the biogenesis of the secretory granules or, alternatively, by an alteration of the directionality of the secretory pathway in the basolateral regions where exocytosis is not favored. (23) The same author had already reported in 2008 the loss of cell polarity at the secretory acini (24).

SNAREs are transmembrane proteins which also participate in the implement of exocytosis mechanism, their position in physiological conditions is at the apical portion.

In 2012 María-José Barrera *et al*. have highlighted: a decrease in MUC5B presence in the labial salivary glands in patients with pSS, and significant alterations in the expression of tight junction proteins both in apical and basolateral cell side. Furthermore, their studies have shown that SNAREs proteins, in Patients with pSS, are positioned at the basal portion, with the consequent exocytosis of mucins (MUC7 and MUC5B) to the extra-cellular matrix (25). The same authors, showed that RAB3D loses it is specific apical localization, (in over 50% of the glandular acini) (26).

Toll-like receptors (TLR) are transmembrane receptors involved in innate immunity and as a trigger to the specific immune response. These receptors are stimulated by molecular patterns of bacteria's and fungi’s cell wall, by DAMPs (damage-associated molecular patterns) produced by cells or by the extracellular matrix damage. by products caused by infections, cell damage and by non-physiological cell death.

In 2015 Marıa-Jose´ Barrera and her team demonstrated that mucins, released to the extra-cellular matrix induce the expression of inflammatory cytokines, where in this inflammatory response TLRs plays a fundamental role.

In their study, the authors claim that alterations in cell polarity lead to the loss of the barrier function of the epithelium, triggering a series of consequent alterations that cause the release of mucins into the extra-cellular matrix. MUC5B mucins and MUC5B saccharide residues are recognized by TLR4 of the epithelial cells, resulting in the initiation of a proinflammatory response. These signals, initially produced by epithelial cells, could attract inflammatory cells, and thus perpetuate inflammation and the development of chronic disease (27).

In 2020 Castro I confirms that there is an intracellular accumulation of MUC7 (17,22) and that the sialylation rate is decreased, probably due to genes’ down-regulation (18). The changes would be a consequence of cytokines’ pro-inflammatory action that induced by anomalous mucins secretion due to loss of basoapical directionality, involved in the secretory chemotactical (20). According to the author, the MUC1, MUC7 and MUC5B glycoproteins excreted to the intercellular space have a chemotactical function, recalling inflammatory and pro-inflammatory molecules and activate the Toll-like receptors that also participate in the inflammatory reaction.

## Discussion

- Saliva Rheological Properties and Mucin Glycosylation

From studies in the literature related to the cause of pSS’s clinical aspects, some common dogmas are questioned. The MUC7 and MUC5B glycoproteins have a pathological reduction of the total sulphate oligosaccharides , (9,14,15,24) and a reduction of the glycidic component, in particular the sialic acid, unlike for the MUC1, where only a quantitative reduction in certain areas of the oral cavity has been reported (16,19).

The qualitative anomalies of the mucins lead to a reduction in the ability of the molecule to bind water and therefore to guarantee an adequate hydration of the mucous membranes.

The reason for the previously mentioned anomalies is not yet completely known, it was first hypothesized the role of sulfurization (14) and then it was demonstrated the connection with reduced mucins glycosylation due to functional anomalies in post-translational compartments, such as endoplasmic reticulum and GC. On a different front, quantitative anomalies of MUC7’s RNAm have not been reported, (9) but a down-regulation of the glycosyltransferase and glycosidase genes, involved in the glycosylation of MUC7 has been demonstrated by miRNA studies (18). These studies would confirm why, in Patients with pSS, in the first phase of the disease, there is a normal quantity of mucin, but qualitatively, mucins result abnormal (18). The down-regulation was demonstrated also by Culp for MUC1 (19).

According to Alliende, the presence of mucins in abnormal sites induces a consistent inflammatory response, related, to the pathogenesis of pSS (14).

The studies presented show, although without giving a clear explanation, that the xerostomia, the reduction of spinnbarkeit and residual mucosal saliva are only partially related to the reduction of the USF, pathological alterations of the chemical-physical properties of mucins prevail in the pathogenesis of pSS’s clinical aspects.

- Structural and/or Metabolism modifications of Mucous Acinar Cells in Salivary Glands

Another unclear aspect is why the glandular acini, containing abnormal mucins, have a functionally incorrect directionality of the secretory pathway. In particular, Rab3D proteins and SNAREs, which have an essential role to facilitate exocytosis, in pSS patients, are rarely present at the apex of muciparous cells (14,23). In healthy subjects, the apex of the cell is where normally the granules are being excreted. In patients with pSS on the contrary, Rab3D molecules and SNAREs are positioned at the basolateral portion, which would explain the directional anomaly of mucins excretion in the intercellular space, but it is not known why this happens (23,26).

Further beyond, there have been also reported abnormalities in the extra-cellular compartment: a disorganization of the basal lamina of mucous cells (9,14) alterations in the expression of tight junction proteins at the apical and basolateral side in response to TNF-alpha and IFN-gamma, (24,26), but surely the most interesting data is the role of membrane TLRs in determining the expression of inflammatory cytokines as a reaction to the mucins released in the extra-cellular matrix (27). The latter evidence, for the first time, attempts to correlate intracellular anomalies with the extra-cellular matrix and autoimmune reactions. The same opinion was put forward by Castro *et al*. in 2020. He supports the hypothesis that there is a cross regulation between cytokines and mucins. The same MUC1, MUC7 and MUC5B excreted to the intercellular space would have a pro-inflammatory initiation function, causing the release of inflammatory molecules and the recruitment immune cells, by activating the Toll-like receptors of the epithelium cells. This process would lead to pericellular and intracellular anomalies in LSG. To this date however, the exact nature of these anomalies have not been clarified (20).

- Th17 reaction and Structural and/or Metabolism modifications of Mucous Acinar Cells in the Salivary Glands and modifications Saliva Rheological Properties

pSS is an autoimmune disease supported by Th17 response. However, so far it is not clear how the immune system acts on LSG cells. According to Verstappen *et al*. the role of IL-17 is fundamental, it usually has a tissue protection function, but in response to environmental stimuli it can trigger an autoimmune reaction with the production of autoantibodies (28). Environmental factors could be MUC7, MUC5B, MUC1 and their saccharide residues; these molecules, excreted to the intercellular space for not completely clear reasons, stimulate the secretion of inflammatory and pro-inflammatory molecules and activate the Toll-like receptors that also participate in the inflammatory reaction, however, a correlation between cytokines and MUC would only partially explain the etiopathogenesis of pSS.

In any case, the resulting Th17 response promotes a chronic inflammatory process in the muciparous glands. IL-17 and its mRNA were detected in the MSG of patients with pSS, in these patients the number of Th17 inflammatory cells and the quantity of IL-17 mRNA are correlated with a greater presence of germinal centers in the glandular interstitium, according the classification of Chisholm DM and Mason DK (29).

Despite the various unclear questions, much evidence suggests that pSS causes qualitative alterations of the rheological properties of the saliva and that these are the main reason, more than the reduction of the USF, for the principal clinical aspects of the disease, dry mouth and complications in the oral cavity; therefore we propose to add a qualitative test of the saliva glycoproteins to the classification criteria of pSS.

## Conclusions

In pSS, especially in the initial phase of the disease, pathological alterations of the chemical-physical properties of the mucins prevail the quantitative alterations, moreover, there are structural and metabolism modifications of the muciparous cells of the salivary glands.

The American College of Rheumatology / European League indicate Sialometry as one of the diagnostic tests of the disease, our opinion is that it would be useful to add to the 5 classification criteria of the pSS also a qualitative test of saliva such as the search for sulfated oligosaccharides or simply the sulfurization of mucins.

It is only now that research has begun to drive a the correlation between Th-17 reaction, muciparous cell anomalies, and modification of the MUC7 and MUC5B glycoproteins, and we are confident that more direct evidence will emerge in the near future.
